# Differential Proteomic Analysis Predicts Appropriate Applications for the Secretome of Adipose-Derived Mesenchymal Stem/Stromal Cells and Dermal Fibroblasts

**DOI:** 10.1155/2018/7309031

**Published:** 2018-08-06

**Authors:** Stefania Niada, Chiara Giannasi, Alice Gualerzi, Giuseppe Banfi, Anna Teresa Brini

**Affiliations:** ^1^IRCCS Istituto Ortopedico Galeazzi, Milan, Italy; ^2^Department of Biomedical, Surgical and Dental Sciences, Università degli Studi di Milano, Milan, Italy; ^3^Laboratory of Nanomedicine and Clinical Biophotonics, IRCCS Fondazione Don Carlo Gnocchi, via Capecelatro 66, 20148 Milan, Italy; ^4^Vita-Salute San Raffaele University, via Olgettina 58, 20132 Milan, Italy

## Abstract

The adult stem cell secretome is currently under investigation as an alternative to cell-based therapy in regenerative medicine, thanks to the remarkable translational opportunity and the advantages in terms of handling and safety. In this perspective, we recently demonstrated the efficient performance of the adipose-derived mesenchymal stem/stromal cell (ASC) secretome in contrasting neuroinflammation in a murine model of diabetic neuropathy, where the administration of factors released by dermal fibroblasts (DFs) did not exert any effect. Up to now, the complex mixture of the constituents of the conditioned medium from ASCs has not been fully deepened, although its appropriate characterization is required in the perspective of a clinical use. Herein, we propose the differential proteomic approach for the identification of the players accounting for the functional effects of the cell secretome with the aim to unravel its appropriate applications. Out of 967 quantified proteins, 34 and 62 factors were found preponderantly or exclusively secreted by ASCs and DFs, respectively. This approach led to the recognition of distinct functions related to the conditioned medium of ASCs and DFs, with the former being involved in the regulation of neuronal death and apoptosis and the latter in bone metabolism and ossification. The proosteogenic effect of DF secretome was validated *in vitro* on human primary osteoblasts, providing a proof of concept of its osteoinductive potential. Besides discovering new applications of the cell type-specific secretome, the proposed strategy could allow the recognition of the cocktail of bioactive factors which might be responsible for the effects of conditioned media, thus providing a solid rationale to the implementation of a cell-free approach in several clinical scenarios involving tissue regeneration.

## 1. Introduction

Adult stem cell-based therapies have been proven effective in resolving a wide array of clinical questions, opening the way to their translation from preclinical models to medical practice. Up to date, 301 clinical trials explored or are currently investigating the safety and performance of mesenchymal stem/stromal cells (MSCs), a class of adult stem cells that can be conveniently harvested from several tissue sources (source: http://clinicaltrials.gov, applied filters: Active, not recruiting + Terminated + Completed as Recruitment Status, Interventional (Clinical Trial) as Study Type). Currently, the therapeutic effect of MSC administration has been tested for the treatment of numerous acute and chronic pathologies, spanning from cardiovascular disorders to musculoskeletal and immune diseases [1, 2]. In the last few years, it has become increasingly evident that the beneficial action exerted by MSCs in these heterogeneous clinical scenarios largely depends on paracrine mechanisms rather than being a direct consequence of cell engraftment [3–9]. The therapeutic potential of the secretome of these cells is currently under investigation, gathering growing consensus because of the remarkable translational ability of the cell-free approach that presents substantial advantages over cell therapy, especially in terms of handling and safety. In the context of tissue regeneration, the secretome of MSCs from bone marrow, adipose tissue, and Warton jelly has been proven effective in preclinical models of Parkinson's disease, spinal cord injury, and ischemic stroke [10, 11], while the one from human umbilical cord mesenchymal stem/stromal cells has been proven effective in ameliorating kidney damage and regenerating atrophied muscles [12, 13]. Moreover, the effects of conditioned medium (CM) from cultured MSCs have been largely explored in multiple biological processes linked to clinically significant events, such as wound healing [14, 15], inflammation blunting [16, 17], angiogenesis [18, 19], and neuropathic pain [8]. In this scenario, we recently demonstrated the therapeutic effect of the administration of the CM from adipose-derived stem/stromal cells (ASCs) in a mouse model of diabetes mellitus, providing a solid evidence of its efficiency in contrasting neuropathic pain, neuroinflammation, and peripheral immune activation [20]. Interestingly, we also established that the observed effects were specifically linked to the cell source, as the treatment with CM derived from dermal fibroblasts (DFs) did not counteract the monitored symptoms.

Considering the therapeutic potential of the cell secretome, we believe that an appropriate characterization is required in the perspective of a clinical use. Since it is widely accepted that the efficacy of the cell secretome is not linked to a single “ingredient” but depends on a cocktail of factors acting in synergy, we are currently characterizing the CM content, in terms of both soluble components and vesicular cargos, by multiple approaches. Recently, we demonstrated that the extracellular vesicles released by different MSCs (i.e., ASCs and MSCs from bone marrow) and DFs possess peculiar features that allow their discrimination through Raman spectroscopy with an accuracy of 93.7% [21]. In our previous *in vitro* and *in vivo* works, DFs were chosen as the term of comparison for MSCs as these cell populations present some common features, such as stromal localization, phenotypic profile, and multilineage differentiative capabilities. Nevertheless, MSCs and DFs differ for important characteristics, among which the distinct anti-inflammatory and angiogenic potential are particularly interesting in the perspective of their employment in the regenerative medicine field [22, 23]. Here, we compared the secretome from ASCs to the one from DFs through a differential proteomic approach, focusing on its potential to predict the action of CM deriving from distinct cell sources on different targets and pathological conditions. We examined the factors differentially expressed between the two populations that may be involved in the antineuroinflammatory properties of ASCs observed *in vivo*. Moreover, on the basis of the factors preponderantly released by dermal fibroblasts, we hypothesize a proosteogenic effect of CM-DFs that was validated *in vitro* with human primary osteoblasts.

## 2. Materials and Methods

Unless otherwise stated, reagents and chemicals were purchased from Sigma-Aldrich (Saint Louis, MO, USA).

### 2.1. Cell Culture

All the cell types used in this study were isolated from waste tissues of healthy donors undergoing plastic (abdominoplasty and liposuction) or orthopaedic surgery, after written consent and following the procedure PQ 7.5.125, version 4, dated 2015-01-22, approved by the IRCCS Galeazzi Orthopaedic Institute. ASCs were isolated from the subcutaneous adipose tissue of 3 female donors (age range: 26–65 y/o) while DFs were isolated from the deepidermised dermis of 3 female patients (age range: 26–46 y/o). Cells were isolated following previously described protocols [21]. Briefly, ASCs were isolated from adipose tissue samples following digestion with 0.75 mg/ml type I collagenase (250 U/mg, Worthington Biochemical Corporation, Lakewood, NJ, USA) and filtering of the stromal vascular fraction. DFs were obtained from fragmented dermis after digestion with 0.1% type I collagenase. Osteoblasts were isolated from the cancellous bone of a female patient (66 y/o) undergoing total hip replacement surgery. Briefly, bone fragments were excised and minced with a scalpel, washed several times in phosphate buffered saline (PBS), and vortexed at high speed in order to remove residual adipose and/or hematopoietic tissue. Bone chips were then plated in petri dishes until cell outgrowth. All cell types were maintained in a humidified atmosphere at 37°C, 5% CO_2_ in complete culture medium (DMEM, 2 mM L-glutamine, 50 U/ml penicillin, 50 *μ*g/ml streptomycin) added with 10% FBS (EuroClone, Milan, Italy). The medium was replaced every other day and, at 70–80% confluence, cells were detached with 0.5% trypsin/0.2% EDTA, plated at a density of 10,000 cells/cm^2^ for ASCs and OBs, 5000 cells/cm^2^ for DFs, and expanded.

### 2.2. Conditioned-Media Production

Conditioned medium was prepared as previously described [20]. Once at 80–90% confluence, cells at the 4th passage were washed twice with PBS, kept for one hour in serum-free, phenol-free complete DMEM, and cultured in the same starving conditions for 72 hours. Conditioned media were then centrifuged at 2500*g* for 15 minutes to remove cell debris and concentrated using Amicon® Ultra-15 centrifugal filter columns with a 3 kDa molecular weight cutoff (Merck Millipore, Burlington, MA, USA). Protein concentration was measured by a Bradford assay (Bio-Rad Laboratories, Hercules, CA, USA).

### 2.3. nLC-MS/MS Analysis and Bioinformatics

Conditioned media samples were delivered to ProMiFa (Protein Microsequencing Facility, San Raffaele Scientific Institute, Milan, Italy) to perform nLC-MS/MS analysis. 20 *μ*g of total proteins from each sample were in-solution digested using the Filter Aided Sample Preparation (FASP) protocol as reported in literature [24]. Aliquots of the samples containing tryptic peptides were desalted using StageTip C18 (Thermo Fisher Scientific, Waltham, MA, USA) and analysed by nLC-MS/MS using a Q-Exactive mass spectrometer (Thermo Fisher Scientific) equipped with a nanoelectrospray ion source (Proxeon Biosystems, Odense, Denmark) and a nUPLC Easy-nLC 1000 (Proxeon Biosystems). Peptide separations occurred on a homemade (75 *μ*m i.d., 12 cm long) reverse phase silica capillary column, packed with 1.9 *μ*m ReproSil-Pur 120 C18-AQ (Dr. Maisch GmbH, Germany). A gradient of eluents A (distilled water with 0.1% *v*/*v* formic acid) and B (acetonitrile with 0.1% *v*/*v* formic acid) was used to achieve separation (300 nl/min flow rate), from 2% B to 40% B in 88 minutes. Full-scan spectra were acquired with the lock-mass option, resolution set to 70,000 and mass range from *m*/*z* 300 to 2000 Da. The ten most intense doubly- and triply-charged ions were selected and fragmented. All MS/MS samples were analysed using the Mascot (version 2.6, Matrix Science) search engine to search the human_proteome 20171122 (93,786 sequences; 37,178,108 residues). Searches were performed with the following settings: trypsin as proteolytic enzyme; 2 missed cleavages allowed; carbamidomethylation on cysteine as fixed modification; protein N-terminus-acetylation, methionine oxidation as variable modifications; and mass tolerance was set to 5 ppm and to 0.02 Da for precursor and fragment ions, respectively. To quantify proteins, the raw data were loaded into the MaxQuant software version 1.5.2.8 [25]. Label-free protein quantification was based on the intensities of precursors. The experiments were performed in technical triplicates. The complete dataset of identified and quantified proteins, as obtained by proteomic analysis, was subjected to Student's *t*-test in order to define significantly differently expressed proteins with a *p* value < 0.05, followed by hierarchical clustering analysis, using MeV software v. 4_9_0 [26].

### 2.4. *In Vitro* Functional Analysis of CM-ASCs and CM-DFs

#### 2.4.1. Cell Viability

3 × 10^3^ osteoblasts/cm^2^ were plated in triplicate on 96-well plates and maintained in culture for 16 days in complete culture medium. At days 2, 4, 7, 9, 11, and 14, cells were treated with CM deriving from 10,000 ASCs or DFs (recipient to donor cell ratio 1 : 10) and viability/proliferation was monitored through time as previously described [27]. Briefly, at each time point the culture media were replaced with the addition of 10% alamarBlue® (Thermo Fisher Scientific, Waltham, MA, USA) and cells were incubated for 3.5 hours at 37°C in the dark. 100 *μ*l of supernatant was then transferred to black-bottom 96-well plates and fluorescence (540 nm excitation *λ*, 600 nm emission *λ*) was read with a Wallac Victor 2 plate reader (PerkinElmer, Waltham, MA, USA). At the end of the experiment, cells were fixed and stained by Diff-Quik, following standard protocol (Medion Diagnostics, Miami, FL, USA). Statistical analysis was performed by two-way ANOVA and Bonferroni's multiple comparison test using GraphPad Prism 5 Software (San Diego, CA, USA).

#### 2.4.2. Real-Time PCR

OBs were plated at a density of 6000 cells/cm^2^ and cultured in standard conditions until confluence. Then, cells were treated with conditioned medium deriving from 120,000 ASCs or DFs (recipient to donor cell ratio 1 : 5). After 24 hours, the expression of osteogenic marker genes (*RUNX2*, *SPP1*, and *COLL I*) and of *VEGF* (recently identified as a fundamental factor involved in bone repair and regeneration [28]) was assessed by real-time polymerase chain reaction (RT-PCR, StepOne Plus, Life Technologies, Carlsbad, CA, USA) [29]. Briefly, total RNA was purified using an RNeasy Mini Kit (Qiagen, Hilden, Germany). cDNA was obtained by a high-capacity cDNA reverse transcription kit and amplified by Single Tube TaqMan® Gene Expression Assays (*RUNX2*: hs00231692_m1, *SPP1*: hs00959010_m1, *VEGF*: hs00959010_m1, and *COLL I*: hs01076777_m1) (Applied Biosystems, Foster City, CA, USA). Data were normalized on *ACTB* (Hs01060665_g1) and the relative quantification was determined using the delta delta CT (ΔΔCT) method.

#### 2.4.3. Western Blot

OBs were seeded at a density of 8000 cells/cm^2^ and cultured in standard conditions until confluence. Cells were treated with conditioned medium deriving from 400,000 ASCs or DFs (recipient to donor cell ratio 1 : 5) for 72 hours. Cells were then lysed in 65 mM Tris-HCl, pH 6.8, with 2% sodium dodecyl sulfate (SDS) supplemented with protease-inhibitor cocktail. 15 *μ*g of whole cell lysates, quantified by BCA Protein Assay (Thermo Fisher Scientific), were resolved into 8% SDS-PAGE and transferred to nitrocellulose membranes (GE Healthcare, Little Chalfont, UK). Membranes were probed overnight with either rabbit polyclonal antibody raised against osteopontin (OPN; Abcam, Cambridge, UK) or mouse monoclonal antibody raised against SPARC (Santa Cruz Biotechnology, Dallas, TX, USA). Goat polyclonal anti-GAPDH antibody (Santa Cruz Biotechnology, Dallas, TX, USA) was employed as a housekeeping protein. Proteins of interest were detected after 45 minutes of incubation with appropriate HRP-conjugated secondary antibodies (Santa Cruz Biotechnology, Dallas, TX, USA) using LiteAblot® Turbo Extra-Sensitive Chemiluminescent Substrate (EuroClone, Milan, Italy). Images were acquired through ChemiDoc Imaging System™ and analysed through Image Lab™ software (Bio-Rad Laboratories, Hercules, CA, USA).

## 3. Results

### 3.1. Differential Secretome Analysis

**Figure 1:**
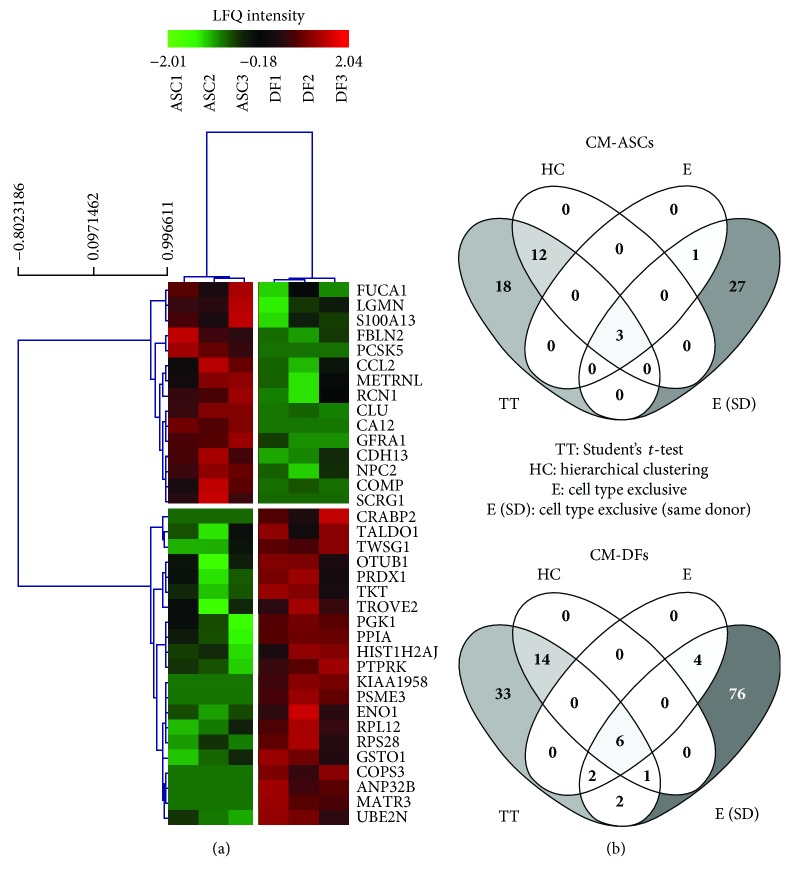
Differential proteome analysis of CM-ASCs and CM-DFs. (a) Hierarchical clustering criteria identify 15 proteins preponderantly secreted by ASCs (*n* = 3) and 21 factors predominantly released by DFs (*n* = 3). The color scale represents the label-free quantification (LFQ) of the relative amount of proteins in the biological samples. (b) Venn diagrams (http://bioinfogp.cnb.csic.es/tools/venny) representing the number of differentially expressed proteins identified by distinct statistical analyses (Student's *t* test alone (TT) or followed by hierarchical clustering (HC)) and discrimination criteria (factors uniquely present in the CM of one cell type considering all samples (E) or considering only ASCs and DFs harvested from the same donor (E (SD)).

We performed a nLC-MS/MS analysis to identify differentially secreted proteins between ASCs and DFs. Three conditioned media of each cell type were analysed. 1208 proteins were identified, 976 of which were quantified. Following a hierarchical clustering approach, we identified two groups of factors that were differently secreted among ASCs and DFs (Figure 1(a)). The results showed that 15 proteins were uniquely or preponderantly present in CM-ASCs, while 21 were present in those from DFs. Using this list as input in the STRING platform [30], it resulted in the majority of differently secreted proteins in both groups (9 for ASCs—FDR: 0.00433 and 15 for DFs—FDR: 4.66*e* − 07) being associated to extracellular exosomes. No relevant functional pathway was associated to these proteins, even though DF-specific factors appeared to be involved in sugar metabolism (monosaccharide biosynthetic process—FDR: 3.47*e* − 05, xylulose biosynthetic process—FDR: 0.00461, and nonoxidative branch of pentose-phosphate shunt—FDR: 0.023).

**Figure 2:**
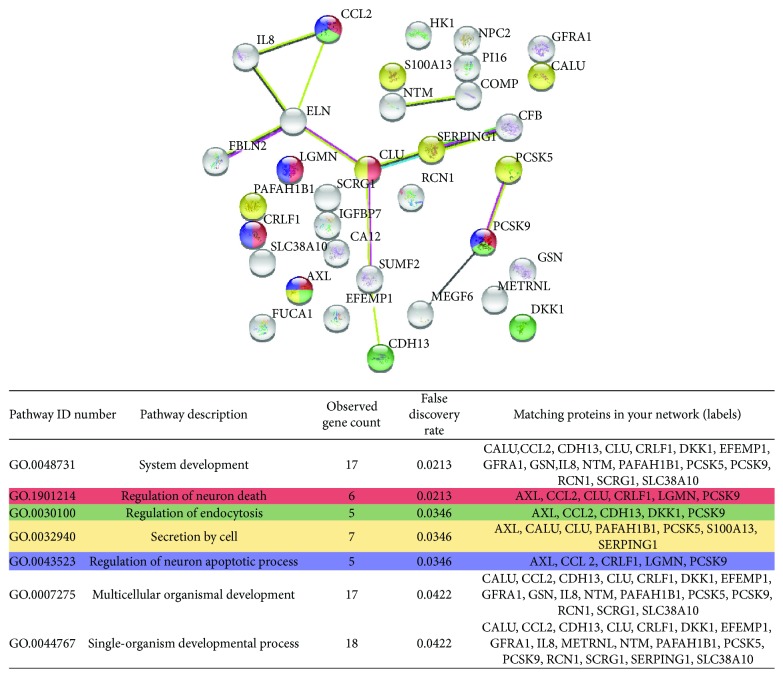
STRING analysis uncovering protein-protein interactions and biological processes associated to the 34 proteins solely or preponderantly secreted by ASCs.

**Figure 3:**
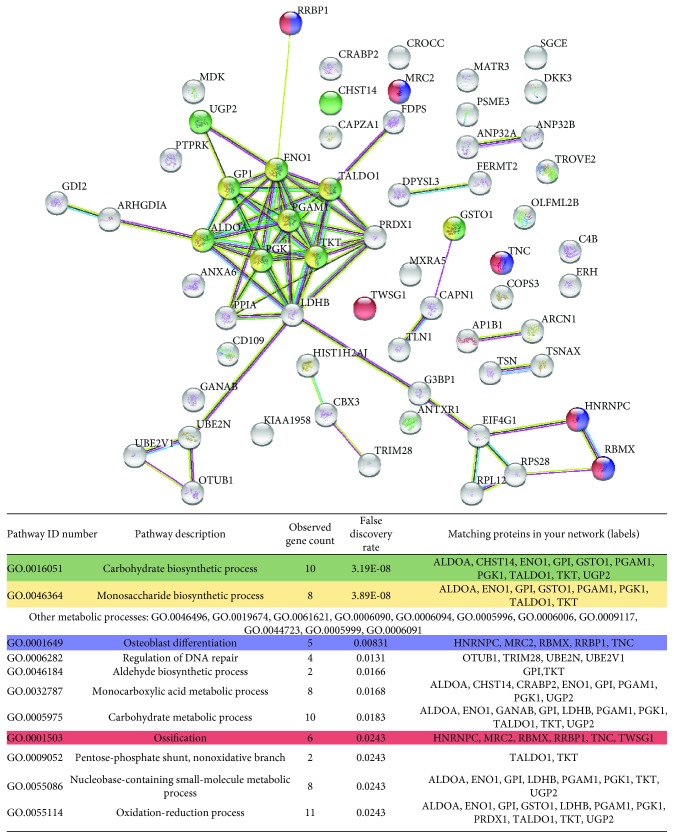
STRING analysis uncovering protein-protein interactions and biological processes associated to the 62 proteins solely or preponderantly secreted by DFs.

Despite that no link to reported pathways was identified for the 15 ASC-specific proteins, several of them have a known immune function (S100 A13 protein, CCL2, LGMN, FUCA1, and METRNL) and/or are involved in neuron death and apoptosis (CCL2, CLU, LGMN, and PCSK5). To widen our analysis and deepen our understanding of the differentially secreted proteins, we applied different criteria. At first, we manually checked the data to identify factors exclusively secreted by either ASCs or DFs. Among the proteins released exclusively by one cell type (4 and 12 for ASCs and DFs, resp.), some factors were not identified through the previous clustering (1 for ASCs and 6 for DFs) (Figure 1(b)). Moreover, applying Student's *t* test with no further correction, we identified additional factors differentially secreted from ASCs (*n* = 18) and DFs (*n* = 35). Finally, we compared the CM of same-donor ASCs and DFs and the factors released uniquely by one population were manually selected. These data confirmed that the two cell types peculiarly release a multitude of different factors (31 ASC-specific proteins and 91 DF-specific proteins), regardless of the donor features. However, since many proteins were solely selected with this criterion (Figure 1(b)), they were not included in further analyses. Considering the Student's *t* test results combined with cell type-exclusive data, we obtained novel and more comprehensive lists accounting for 34 (Figure 2) and 62 (Figure 3) factors differentially or exclusively secreted by ASCs and DFs, respectively.

These multiple proteins were run in a novel STRING analysis. Functional enrichment confirmed that the conditioned media of the two cell types contained many different proteins associated to extracellular vesicles (17 proteins for ASCs—FDR: 3.55*e* − 05 and 34 proteins for DF—FDR: 7.84*e* − 13). Moreover, it reinforced the previous observation that CM-DFs contain proteins involved in metabolic processes (Figure 3) and suggested that in CM-ASCs there are different factors regulating endocytosis and cell secretion (Figure 2). Regeneration functions associated to cell type-specific factors were also identified. In particular, the regulation of neuron death and of neuronal apoptotic properties were among the first 5 results regarding biological processes associated to ASC factors (Figure 2), consistently with our preclinical data on neuropathic pain [20]. Differently, beside sugar metabolism, several DF factors appeared to have a role in ossification and/or bone metabolism (HNRNPC, MRC2, RBMX, RRBP1, TNC, and TWSG1, Figure 3). In order to validate this last observation, we performed functional tests on osteoblasts isolated from a human bone specimen.

### 3.2. Effects of ASC and DF Secretome on Human Primary Osteoblasts

**Figure 4:**
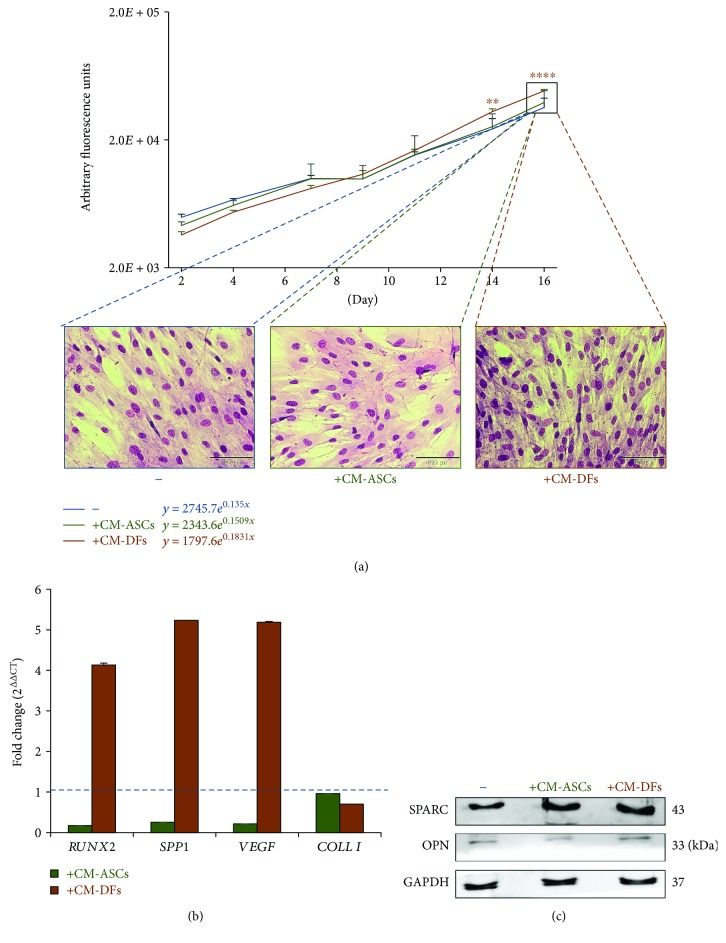
*In vitro* effect of CM-ASCs and CM-DFs on osteoblasts. (a) Semilog graph representing osteoblast viability assessment at different time points of untreated cells (blue line), cells treated with CM-ASCs (green line), or cells treated with CM-DFs (orange line). The equations of the exponential functions describing osteoblast growth in the different conditions are shown. Data represents the mean ± SD of 3 replicates (difference versus untreated ^∗∗^ *p* < 0.01 and ^∗∗∗∗^ *p* < 0.0001). Microphotographs are representative of cell confluence for each group at the final time point. Scale bars = 100 *μ*m. (b) Relative expression of important osteoblast genes after 24 hours of treatment with CM-ASCs (green bars) or CM-DFs (orange bars) in respect to untreated cells, by RT-PCR. The mRNA levels of Runt-related transcription factor 2 (*RUNX2*), osteopontin (*SPP1*), vascular endothelial growth factor (*VEGF*), and type I collagen (*COLL I*) are represented in relation to *β*-actin, here used as a internal control. Data are shown as mean ± SD of technical duplicates. (c) Protein expression of extracellular matrix components by osteoblasts treated with CM-ASCs or CM-DFs for 72 hours. Western blot analysis of osteonectin (SPARC) and osteopontin (OPN) expression following the treatments. Bands were quantified by densitometry and normalized on GAPDH.

As a proof of concept, the involvement of DF-specific factors in bone metabolism/ossification was validated by testing the effects of the secretome of same-donor (46 y/o female donor) ASCs and DFs, on cultured human primary osteoblasts. At first, we investigated the influence of CM treatments on osteoblast viability over a period of two weeks (Figure 4(a)). While the effect of CM-ASCs was almost undetectable, CM-DFs strongly stimulated osteoblast viability through time and the lag phase observed between days 7 and 9 in other groups was avoided. At day 14, we observed a larger number of osteoblasts treated with CM-DFs with respect to other groups (Figure 4(a), microphotographs), demonstrating that this treatment favored cell proliferation rather than enhancing cell metabolism. Then, the short-term effect of the secretome on gene expression was investigated (Figure 4(b)). After 24 hours of treatment, the levels of *RUNX2*, *SPP1*, and *VEGF* mRNA resulted in an enhancement by CM-DFs (fold change of 4.1 for *RUNX2* and 5.2 for both *SPP1* and *VEGF*), thus supporting our hypothesis of a proosteoblastic action of CM-DFs. By contrast, CM-ASCs reduced the expression of these genes of about 4 times while *COLL I* expression was slightly diminished by both treatments. Finally, we investigated the effect of a longer CM treatment (72 hours) on the intracellular protein levels of SPARC and OPN (Figure 4(c)), two important extracellular matrix components. The stimulating effect of CM-DFs on osteoblasts was confirmed by a +123% increase in SPARC expression with respect to untreated cells. However, a slightly minor upregulation of SPARC was also induced by CM-ASCs. In addition, a little increase (+29%) in the naïve 33 kDa form of OPN [31] was also ascribable to the DF secretome only. Nevertheless, this effect was not maintained for all the protein isoforms subject to posttranslational modifications (data not shown).

## 4. Discussion

In light of the fact that conditioned media could be used in the future as biotechnological products for regenerative medicine, it is essential to carry out their characterization before proceeding to clinics. Here, we have implemented differential proteomics to investigate the secretome of ASCs and DFs, focusing on the potential applications in the regenerative medicine field. We applied different criteria to select proteins that were differentially or exclusively released by one cell type and we came up with 34 and 62 factors uniquely or prevalently secreted by ASCs and DFs, respectively. The STRING analysis of these factors led to the recognition of distinct functions related to the CM of ASCs and DFs which are consistent with previous findings on ASC-CM neuroprotective effects [20, 32–34] and with our current results on DF secretome osteoinductive properties (Figure 4). Several proteins released more abundantly or exclusively by ASCs, namely AXL, CCL2, CLU, CRLF1, LGMN, and PCSK9, were found to be significantly associated with the regulation of neuronal death and apoptosis. These bioactive factors might contribute to the modulation of neuroinflammation exerted by CM-ASCs in our preclinical models of neuropathic pain [20, 35] by activating different mechanisms. AXL is a receptor tyrosine kinase which regulates the innate immune system activation [36] and controls the phagocytosis of dead neurons. Even though this protein is probably released by ASCs through exosomes (STRING, Exocarta), it still needs to be shown whether it is transferred to recipient cells. Chemokine (C-C motif) Ligand 2 (CCL2) is a pleiotropic chemokine with an important role in neurogenesis exerted by promoting glial cell proliferation and growth, inducing stem cell migration into sites of damage, and directing differentiation of precursor cells into neurons, astrocytes, and oligodendrocytes [37]. Clusterin (CLU) is a stress-induced chaperone involved in neuronal protection [38]. Its levels are increased in multiple degenerative conditions [39] and following traumatic brain injury [40]. CLU modulates neuroinflammation also thanks to its capacity to suppress complement activation. Legumain (LGMN) is a cysteine protease which regulates the development of immune response and tolerance by playing a key role in the processing of antigens [41]. Among its functions, the involvement in the axonal regeneration following spinal cord injury in zebrafish is particularly intriguing [42]. Finally, the soluble receptor Cytokine Receptor-Like Factor 1 (CRLF1) and the secreted protease Proprotein Convertase Subtilisin/Kexin type 9 (PCSK9) may contribute to neuroprotection due to their activity in promoting neuronal cell survival [43] and in regulating neuronal apoptosis [44], respectively. In addition, two factors specifically released only by ASCs could contribute to the therapeutic action of their CM. Indeed, similarly to CCL2, CXCL8/IL-8 is known to possess neuroprotective features [37]. Furthermore, Scrapie Responsive Gene 1 (SCRG1), which is a positive regulator of stem cell self-renewal, migration, and differentiation potential [45], has been recently proposed as an inhibitor of the infiltration of monocytes, dendritic cells, natural killer cells, and chronically activated T lymphocytes [46]. Other proteins with a known immune function were highlighted by the hierarchical clustering analysis. Among them, Alpha-L-Fucosidase 1 (FUCA1) exerts immunoregulatory actions [47], Meteorin-like protein (METRL) stimulates the expression of anti-inflammatory cytokines [48], and S100 calcium-binding protein A13 (S100A13) acts as a regulator of macrophage inflammation [49]. Our hypothesis is that all these proteins concur in the conversion of the proinflammatory/neurodestructive environment observed in diabetic mice into an anti-inflammatory/neuroprotective one [20]. Further investigations with CM previously deprived of specific factors could confirm their involvement in the neuroinflammation blunting. In any case, our unbiased approach allowed us to identify neuroprotection as one of the main functions of the ASC secretome. The modulation of neurodegenerative and neuroinflammatory diseases by the release of neurotrophic and immunomodulative molecules is well documented not only for ASCs but also for other MSCs [10, 33] and it explains the multitude of applications for MSCs and their conditioned media in these contexts [20, 32, 35, 50, 51].

On the other hand, the analysis of the factors preponderantly or specifically released by DFs produced unexpected outcomes. CM-DFs resulted particularly enriched in proteins involved in sugar metabolism and further investigations should aim at deciphering whether these factors might be involved in the production of glycosaminoglycans and/or in other metabolic processes. Of note, several proteins, which are involved in bone metabolism and ossification (HNRNPC, MRC2, RBMX, RRBP1, TNC, and TWSG1), were also highlighted. Among them, Tenascin C (TNC) is an extracellular matrix glycoprotein implicated in osteoblastic differentiation and mineralization within the bone, probably acting as a mediator of TGF-*β*-induced new bone formation [52]. C-type mannose receptor 2 (MRC2, also known as ENDO180, CD280, or uPARAP) plays a supporting role in bone development being involved in collagen trafficking and deposition [53]. Another factor involved in collagen turnover, particularly in its biosynthesis, is Ribosome-Binding Protein 1 (RRBP1, also known as p180). All these proteins, together with RBMX (RNA-Binding Motif Protein, X-Linked) and HNRNPC (Heterogeneous Nuclear Ribonucleoproteins C1/C2), have been reported to be significantly upregulated during the osteogenic differentiation of MSCs [54], suggesting their role in osteogenesis. Finally, the secreted protein Twisted Gastrulation Homolog 1 (TWSG1) is known to bind bone morphogenetic proteins and to influence osteoblast maturation, even though with contrasting effects [55, 56]. Interestingly, it also inhibits osteoclastogenesis [57], supporting a primary role as a bone-building effector. Since up to now the CM-DF osteoinductive potential has never been reported, we performed a functional *in vitro* test which provided evidence of the proosteogenic function predicted from the proteomic analysis. In this study, CM-DFs switched from a term of comparison, convenient to point out the specific effectors contained in CM-ASCs, to an interesting source of bioactive factors. With our results, we do not want to minimize the proosteogenic potential of the MSC secretome, which has been already documented especially for BMSCs [58], but to suggest novel applications for DFs. In fairness, the potential use of these cells in supporting tissue regeneration has been largely investigated, mainly considering their ability to synthesize and deposit extracellular matrices and release bioactive molecules [59]. Further investigations will be focused on assessing the effects of the DF secretome and/or of its single components on other cell types.

Interestingly, most of the factors differentially released by ASCs and DFs are contained in extracellular vesicles, but only half of the proteins involved in the regenerative functions are released as vesicular cargos (STRING). This remark is consistent with recent observations of a major effect exerted by the whole conditioned medium compared to the exosomes only [60] (our unpublished observations). Further studies investigating this issue are strongly recommended before choosing the proper cell product to be used in specific applications.

## 5. Conclusions

This study provides evidence that the differential proteomic analysis constitutes a useful tool to determine the proper therapeutic target of conditioned media derived from different cell types. Our data reinforced previous observations on the neuroprotective action of the ASC secretome by pointing out specific factors involved in this process and identifying an unexpected proosteogenic aptitude of CM-DFs. This method might be applied to identify the bioactive factors which are released by different cells and are responsible for the biological effect of their conditioned media. At last, a proper validation of specific CM factors could pave the way for the future production of artificial cocktails of bioactive molecules (a novel biological medical product) to be used in different regenerative medicine applications.

## Data Availability

Data Used in the manuscript is available as a Supplementary Materials (available here).

## Supplementary Materials

Supplementary Materials List of 976 quantified proteins. Sample 1 = ASC2; sample 2 = ASC1; sample 3 = ASC3; sample 4 = DF1; sample 5 = DF2; sample 6 = DF3.
